# Weekly Paclitaxel, Carboplatin, and Cetuximab as First-Line Treatment of Recurrent and/or Metastatic Head and Neck Squamous Cell Carcinoma for Patients Ineligible to Cisplatin-Based Chemotherapy: A Retrospective Monocentric Study in 60 Patients

**DOI:** 10.3389/fonc.2021.714551

**Published:** 2021-10-27

**Authors:** Hélène Carinato, Mickaël Burgy, Régine Ferry, Cathie Fischbach, Michal Kalish, Sébastien Guihard, Youssef Brahimi, Henri Flesch, Guy Bronner, Philippe Schultz, Véronique Frasie, Alicia Thiéry, Martin Demarchi, Thierry Petit, Alain C. Jung, Pierre Wagner, Pierre Coliat, Christian Borel

**Affiliations:** ^1^ Institut de Cancérologie Strasbourg Europe, Department of Medical Oncology, Strasbourg, France; ^2^ Centre Paul Strauss, Department of Medical Oncology, Strasbourg, France; ^3^ Institut de Cancérologie Strasbourg Europe, Department of Radiation Oncology, Strasbourg, France; ^4^ Clinique Sainte Barbe, Head and Neck Surgery Unit, Strasbourg, France; ^5^ Hôpitaux Universitaires de Strasbourg, Department of Otorhinolaryngology and Head and Neck Surgery, Strasbourg, France; ^6^ Institut de Cancérologie Strasbourg Europe, Supportive Care Unit, Strasbourg, France; ^7^ Institut de Cancérologie Strasbourg Europe, Department of Biostatistics, Strasbourg, France; ^8^ Institut de Cancérologie Strasbourg Europe, Laboratoire de Biologie Tumorale, Strasbourg, France; ^9^ Université de Strasbourg, Inserm, UMR_S1113, Strasbourg, France; ^10^ Centre Paul Strauss, Department of Radiology, Strasbourg, France; ^11^ Institut de Cancérologie Strasbourg Europe, Pharmacy Department, Strasbourg, France

**Keywords:** head and neck squamous cell carcinoma, recurrent or metastatic, chemotherapy, cetuximab, paclitaxel, carboplatin, first-line

## Abstract

**Objective:**

For most patients suffering from recurrent and/or metastatic head and neck squamous cell carcinoma (R/M HNSCC), chemotherapy is the main option after considering surgery and reirradiation. Cetuximab combined with a platinum-fluorouracil regimen (EXTREME) has been the standard of care for over a decade. Nevertheless, a significant number of patients remain unfit for this regimen because of age, severe comorbidities, or poor performance status. The aim of this study is to investigate an alternative regimen with sufficient efficacy and safety.

**Methods:**

We reviewed retrospectively the medical charts of all patients treated with paclitaxel, carboplatin, and cetuximab (PCC) at our institution. Eligibility criteria were as follows: first-line R/M-HNSCC of the oral cavity, oropharynx, hypopharynx, or larynx not suitable for local therapy, cisplatin, and/or 5-FU ineligibility, ECOG-PS: 0–2. PCC consisted of paclitaxel 80 mg/m^2^, carboplatin AUC 2, and cetuximab at an initial dose of 400 mg/m^2^ then 250 mg/m^2^, for 16 weekly administrations followed by cetuximab maintenance for patients for whom a disease control was obtained. The primary endpoint was overall survival (OS), and secondary endpoints were overall response rate (ORR), progression free survival (PFS), and safety.

**Results:**

We identified 60 consecutive patients treated with PCC between 2010 and 2016 at our institution. Thirty-one patients (52%) were ECOG-PS 2. Fifty-five patients (92%) were cisplatin ineligible. ORR was 43.3% (95% CI, 30.8–55.8), and disease control rate was 65% (95% CI, 52.9–77.1). With a median follow-up of 35.7 months (IQR 28.6–48.8), median PFS was 5.8 months (95% CI, 4.5–7.2), and median OS was 11.7 months (95% CI, 7.5-14.8). For ECOG-PS 0–1 patients, median OS was 14.8 months (95% CI, 12.2–21.7) while it was only 7.5 months (95%CI: 5.5-12.7) for ECOG-PS 2 patients (*p* < 0.04). Grades III–IV toxicities occurred in 30 patients (50%). Most toxicities were hematologic. Six patients (10%) had febrile neutropenia. Nonhematologic toxicities were reported such as cutaneous toxicities, neuropathy, infusion-related reactions, or electrolyte disorders.

**Conclusion:**

The weekly PCC regimen seems to be an interesting option in cisplatin-unfit patients. This study shows favorable PFS and OS when compared with what is achieved with the EXTREME regimen and a high controlled disease rate with predictable and manageable toxicities even in the more fragile population.

## Introduction

Head and neck squamous cell carcinoma (HNSCC) represent more than 90% of the head and neck tumors. In Europe, approximately 140,000 new cases were diagnosed in 2014, corresponding to an annual incidence of 43/100,000. The main risk factors of HNSSC are tobacco with alcohol heavy/frequent consumption and HPV infection (1). In France, most patients are active or former smokers frequently in association with a high consumption of alcohol. Thus, they are likely to suffer from several active tobacco/alcohol-related comorbidities, undernourishment, and other active carcinomas. Considering significant concomitant nonmalignant diseases, age and general condition are crucial in oncological decision-making because a vast majority of patients turns out to be ineligible for clinical trial or standard of care (2).

Concerning recurrent and/or metastatic squamous cell carcinoma of head and neck (R/M HNSCC), palliative chemotherapy is the standard of care if local treatment (surgery or radiotherapy) cannot be curative. There is a need to find an optimal strategy to achieve the highest possible overall survival and patient’s quality of life. In 2008, the EXTREME trial (3) showed the benefits of adding cetuximab to a platinum-5-FU chemotherapy in R/M HNSCC first-line treatment. An overall response rate (ORR) of 36% was achieved, median progression free survival (PFS) was 5.6 months, and median overall survival (OS) was 10.1 months. The EXTREME regimen has emerged as the standard of care for fit R/M HNSCC patients. Nevertheless, numerous patients remain unfit for this regimen because of frailties such as age, ECOG-PS >1, or heavy comorbidities, as evidenced by 82% of grades III–IV toxicities (3).

Other treatments consisting of a platinum-based chemotherapy associated with taxanes (docetaxel or paclitaxel) were investigated for HNSCC including R/M HNSCC (4). In a phase II trial (5), docetaxel combined with a cisplatin and cetuximab regimen (TPEx) achieved promising outcomes with a 44.4% ORR and a 79.6% disease control rate (DCR). Thus, the same authors carried out a phase II randomized trial (TPExtreme), comparing TPEx with EXTREME in terms of efficacy and safety (6). Results suggest that taxanes are an option in first-line treatment. However, this regimen should be used exclusively in cisplatin fit patients.

Therefore, some studies investigated alternative polychemotherapies in nonfit patients. Carboplatin with weekly paclitaxel is a safe and recommended option in the elderly population affected by advanced nonsmall cell lung cancer (7). In R/M HNSCC as well, some smaller nonrandomized studies demonstrated that first-line weekly carboplatin and paclitaxel could be safely used and improved efficacy when compared with monotherapy schedules in unfit patients (8). The paclitaxel, carboplatin, and cetuximab (PCE) regimen showed a 40% ORR, 5.2 months median PFS, and a 14.7-month median OS as first-line treatment in R/M HNSCC patients (9). The weekly paclitaxel, carboplatin, and cetuximab (PCC) regimen was first reported with promising results by Kies et al. in the locally advanced setting with a high dose of paclitaxel (10). The aim of this study is to provide a deeper insight into the weekly PCC efficacy and safety in the first-line R/M setting.

## Patients and Methods

### Patient Selection

We retrospectively reviewed data from medical charts at our institution (Centre Paul Strauss, Strasbourg, France) between January 2010 and December 2016 to identify patients treated with paclitaxel, carboplatin, and cetuximab and suffering from histologically confirmed SCC of the oral cavity, oropharynx, larynx, hypopharynx, or cervical lymph node from assumed HNSCC. Patients with skin SCC and sinus or nasopharynx carcinoma with poor differentiation were not selected.

Adult patients (aged 18 or older) in first-line treatment of a metastatic and/or recurrent HNSCC with no curative intent, ECOG-PS 0-2, and cisplatin and/or 5-FU ineligibility were included. Patients were considered cisplatin ineligible if at least one of the following criteria was met: age ≥70 or ECOG-PS 2 or creatinine clearance <60 ml/min or significant active comorbidities or cisplatin free interval <6 months. Patients were considered 5-FU ineligible in case of severe cardiovascular previous history including coronary insufficiency whether or not complicated by myocardial infarction, heart insufficiency, or lower limb arteriopathy of at least stage II. A treatment by radiotherapy or surgery for a previous locoregional relapse was permitted.

Induction regimen and palliative second-line or further treatment by PCC were excluded of the analysis.

**Figure 1:**
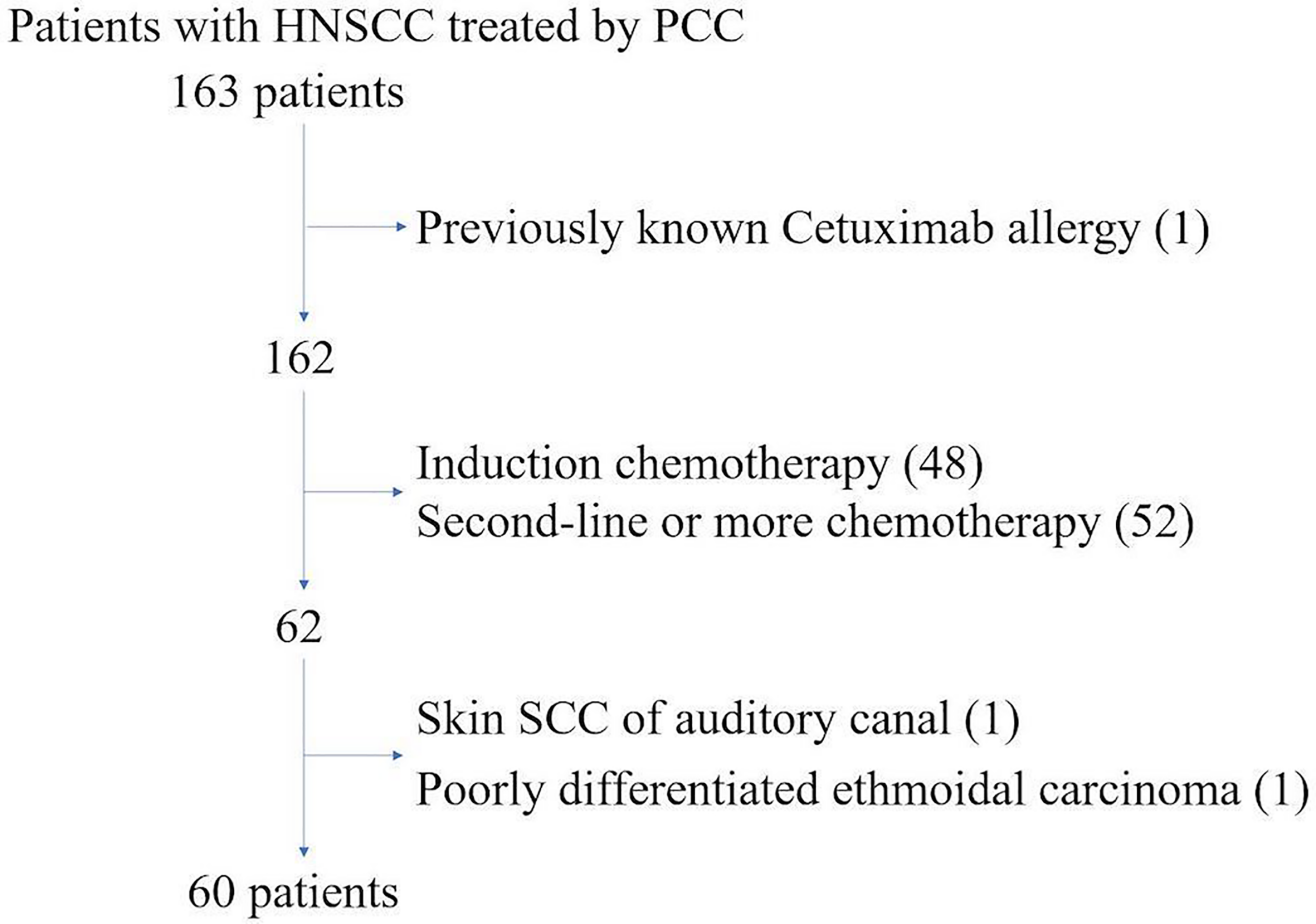
Selection process.

The selection process is shown in **Figure 1**.

### Treatment

From January 2010 to January 2012, we administered the PCC regimen as follows: carboplatin AUC 5 on day 1; paclitaxel 80 mg/m² on days 1, 8, and 15; and cetuximab 400 mg/m² loading dose then 250 mg/m² weekly. This pattern was repeated on day 22. A maximum of six cycles was administrated followed by a cetuximab maintenance given weekly or biweekly until progressive disease or unacceptable toxicities. However, administering carboplatin once every 3 weeks caused hematologic toxicity to such an extent that it became frequently impossible to administer paclitaxel on D15 and even sometime on D8.

Thus, from February 2012 to December 2016, the carboplatin infusion schedule was modified switching to a weekly administration: weekly carboplatin AUC 2 (maximum dose of 220 mg); paclitaxel 80 mg/m²; and cetuximab 400 mg/m² loading dose then 250 mg/m². A maximum of 16 cycles was performed followed by a cetuximab maintenance given weekly (250 mg/m²) or every 2 weeks (500 mg/m²) until progressive disease or unacceptable toxicities.

Doses could be reduced initially or during treatment according to patient’s comorbidities or toxicities.

### Assessment

The efficacy of the protocol was assessed on the basis of response rate, progression-free survival and overall survival. A computed tomographic scan (or 18F-FDG PET/CT if needed) was performed at baseline, then every eight weeks. Measurements were compared between baseline and 8th-week CT scan (or 18F-FDG PET/CT) according to RECIST 1.1 criteria. The ORR is the complete response (CR) rate plus the partial response (PR) rate. The DCR is the ORR plus the stable disease (SD) rate. Radiologically, unevaluable patients were considered progressive if clinical reports mentioned it.

Toxicities were monitored weekly throughout the treatment and evaluated using the Common Terminology Criteria for Adverse Events version 4.0. Dose intensity data were calculated in order to assess regimen feasibility.

### Statistical Analysis

PFS (time from first PCC infusion to progression or death) and OS (time from first PCC infusion to death) were estimated by the Kaplan-Meier method. If progression or death did not occur before the cutoff date, data were censored at the time of the last valid assessment.

The follow-up time is calculated from first PCC infusion to data cutoff (16 Jun 2018).

## Results

### Patient Characteristics

Sixty patients were treated with the first-line combination of paclitaxel and carboplatin plus cetuximab for a R/M HNSCC at our institution between January 2010 and December 2016. Median age was 61, with 10 patients (17%) aged 70 or more. Sex ratio was 5:1. In our study, the main risk factor was tobacco smoking as 80% of patients were former or current smokers. HPV infection was only assessed in two patients with oropharyngeal SCC by using p16 immunohistochemistry as a surrogate marker: one patient was p16 positive, the other one was not.

Five patients were diagnosed with distant metastases at the initial assessment and received PCC as a first treatment. Fifty-five patients had been pretreated with surgery and/or chemoradiotherapy. Fifteen patients in a recurrent setting had received locoregional treatments with a curative intent (such as surgery or reirradiation).

Forty-six patients (77%) were diagnosed with a locoregional relapse, among whom 38 patients in the field of an earlier irradiation. Twenty-nine patients (48%) had been already treated with platinum-based regimen in a neoadjuvant setting (18%) and/or with concurrent radiotherapy (32%) as a multimodality treatment of their initial tumor. Platinum-free interval was less than 3 months in 11 patients (18% of the whole patient population), between 3 and 6 months in three patients (5%) and longer than 6 months in 15 patients (25%). Because of a cisplatin-related kidney failure after a single course of TPEx, one patient received subsequently a PCC regimen. Patient characteristics are summarized in **Table 1**.

**Table 1:** Baseline characteristics of the patients.

Variable		*N* = 60
Gender [*n* (%)]	*n*	60
	Male	50 (83)
	Female	10 (17)
Age (years)	Median	61
	Range	23–79
Age [*n* (%)]	*n*	60
	<65 years	36 (60)
	65–69 years	14 (23)
	≥70 years	10 (17)
ECOG-PS [*n* (%)]	*n*	60
	0	9 (15)
	1	20 (33)
	2	31 (52)
Tobacco status [*n* (%)]	*n*	60
	Nonsmoker	12 (20)
	Current or former smoker	48 (80)
Primary tumor localization [*n* (%)]	*n*	60
	Oropharynx	23 (40)
	Oral cavity	17 (28)
	Hypopharynx	12 (20)
	Larynx	7 (12)
	Unknown	1 (2)
Histologic type [*n* (%)]	*n* (not specified or missing)	38 (22)
	Well differentiated	8 (21)
	Moderately differentiated	23 (60)
	Poorly differentiated	7 (18)
Initial treatment [*n* (%)]	*n*	60
	Neoadjuvant chemotherapy + Surgery	5 (8)
	Neoadjuvant chemotherapy + Surgery + CRT	1 (2)
	Neoadjuvant chemotherapy + CRT (cetuximab)	5 (8)
	Surgery	9 (15)
	Surgery + RT	6 (10)
	Surgery + CRT (platin-based)	15 (25)
	Surgery + CRT (other)	7 (12)
	RT alone	2 (3)
	CRT (cisplatin)	3 (5)
	CRT (cetuximab)	2 (3)
	No prior treatment	5 (8)
Local treatment for first relapse with a curative intent [*n* (%)]	Surgery	11 (18)
	Reirradiation	4 (7)
Tumor extension at baseline [*n* (%)]	*n*	60
	Loco regional only	33 (55)
	Loco regional and metastatic	13 (22)
	Metastatic only	14 (23)
Characteristics of relapse [*n* (%)]	*n*	60
	Relapse in RT field	38 (63)
	Relapse after platinum-based regimen (neoadjuvant, CRT)	29 (48)
Platinum free interval before baseline [*n* (%)]	*n*	29
	<3 months	11 (38)
	3–5.9 months	3 (10)
	≥6 months	15 (52)
Chemotherapy ineligibility [*n* (%)]	*n*	60
	Cisplatin	55 (92)
	5-FU	34 (57)

ECOG PS, Eastern Cooperative Oncology Group Performance Status; CRT, concurrent chemoradiotherapy; RT, radiotherapy.

Twenty-nine patients (48%) were ECOG-PS 0–1 and 31 patients (52%) were ECOG-PS 2 at treatment onset. Frailty characteristics such as undernourishment and active comorbidities are reported in **Table 2**.

**Table 2:** Frailty criteria of patients.

List of frailty criteria [*n* (%)]	*n*	60
	Age >70 years	10 (17)
	ECOG-PS = 2	31 (52)
	Undernourishment^a^	45 (75)
	*Significant active associated comorbidities*	
	Severe atheroma	32 (53)
	Heart insufficiency	10 (17)
	Chronic obstructive lung disease, ≥ stage 2	19 (32)
	Kidney insufficiency	2 (3)
	Pre-existing neuropathy	5 (8)
	Previously cured cancer	17 (28)
	Synchronous active cancer	6 (10)
	Others (psychiatric disorder, cirrhosis, organ transplant, etc.)	35 (58)
Number of criteria [*n* (%)]	*n*	60
	None	1 (2)
	1 criterion	10 (17)
	2 criteria	12 (20)
	3 or more criteria	37 (62)

a Undernourishment: albumin <30 g/L or weight loss over 5% in 6 months or weight loss over 2% if BMI >20 or BMI <18.5 or BMI <21 in 70 years and more aged patients.

As defined in the inclusion criteria, 55 patients (92%) were ineligible to cisplatin. Thirty-four patients (57%) were ineligible to 5-FU because of severe cardiovascular comorbidities.

A second primary cancer arose in six patients during follow-up: two patients with a nonsmall cell lung cancer, one patient with a cutaneous melanoma and a nonsmall cell lung cancer, one patient with a hepatocellular carcinoma, one patient with a cutaneous squamous cell carcinoma, and one patient with a prostate adenocarcinoma.

### PCC Delivery

Among the 60 patients included, six were treated with the first pattern to be used (carboplatin AUC 5 every 3 weeks, weekly paclitaxel 80 mg/m², and cetuximab 400 mg/m^2^ initial dose, followed by weekly 250 mg/m^2^). Starting in 2012, the 54 following patients were treated with weekly carboplatin AUC 2 (maximum dose of 220 mg: 49 patients were involved in dose limiting), paclitaxel 80 mg/m², and cetuximab 400 mg/m² loading dose then 250 mg/m². A maximum of 16 cycles was performed followed by a maintenance administration of cetuximab given weekly (250 mg/m²) or biweekly (500 mg/m²) until progressive disease or unacceptable toxicities.

A first clinical and radiological evaluation was done after eight cycles of PCC. As shown in **Table 3**, seven patients (12%) did not resume chemotherapy due to unacceptable toxicities and 16 patients (27%) because of progressive disease. A focal treatment (neck and/or metastasis) was carried out in seven patients (12%) because of a particularly good partial response. Cetuximab maintenance began after this assessment in eight patients (13%).

**Table 3:** PCC delivery before cetuximab maintenance.

Variable	*N* = 60	Paclitaxel 80 mg/m²/week	Carboplatin AUC2/week	Cetuximab 400 mg/m² then 250 mg/m²/week
Number of cycles	Median	9.5	9.5	10.5
	Range	1–19	1–21	1–21
Early discontinuation of treatment (≤8 cycles):			24 (40)	
- Due to unacceptable toxicities	*n* (%)		7 (12)	
- Due to progressive disease	*n* (%)		16 (27)	
- Change of treatment (local treatment, etc.)	*n* (%)		7 (12)	
Delivery completed (≥16 cycles)	*n* (%)		24 (40)	
Patients with dose reductions	*n* (%)	19 (32)	37 (62)	3 (5)
Patients with ≥1 dose held for ≥7 days	*n* (%)	27 (45)	27 (45)	23 (38)
Dose intensity**^a^**	Median	65	1.6	250
	Range	40–80	0.8–2	125–250

AUC, area under the curve. ^a^Dose delivered per week, accounting for treatment delays and dose reductions. Units of measure are as follows: paclitaxel: mg/m²/week; carboplatin: AUC/week; cetuximab: mg/m²/week. The loading dose of cetuximab (i.e., 400^a^mg/m²) was not included in the calculation of dose density.

Median number of delivered cycles was 9.5 for chemotherapy and 10.5 for cetuximab. Twenty-four patients (40%) completed the 16 cycles of treatment, of whom 17 patients (28%) with the three drugs, while carboplatin or paclitaxel had to be stopped in seven patients.

Doses of paclitaxel and/or carboplatin and/or cetuximab had to be reduced in 19 patients, 37 patients, and three patients, respectively. Forty-five percent of patients experienced delayed chemotherapy due to side effects. PCC had to be stopped in 16 patients (27%) because of severe toxicities.

Toxicities are reported in **Table 4**. Thirty patients (50%) showed grades III–IV toxicities. Most toxicities were hematologic. Blood transfusions were required in 18 patients (30%). EPO and G-CSF were used as secondary prophylaxis in respectively nine (15%) and 30 patients (50%). Sixteen unexpected hospitalizations occurred due to infection, including six febrile neutropenia (10%). Four infections (6.6%), mostly pneumopathies, led to death which occurred only in ECOG-PS 2 patients. No other toxicity brought toxic death about. We observed 15 grades III–IV (25%) nonhematologic toxicities. Hypokalemia and hypomagnesemia are the most noticeable nonhematologic toxic effects in our study.

**Table 4:** Maximal toxicity per patient.

	Grade I-II	Grade III	Grade IV
Overall toxicities [*n* (%)]	21 (35)	30 (50)	
Non hematologic toxicities [*n* (%)]	14 (23)	15 (25)	
Cutaneous	12 (20)	7 (12)	0
Neuropathy	3 (5)	2 (3)	0
Electrolytes disorders	3 (5)	5 (8)	2 (3)
Infusion reaction	3(5)	1 (2)	0
Nausea	5 (8)	0	0
Diarrhea	4 (7)	1 (2)	0
Hematologic toxicities [*n* (%)]	17 (28)	21 (35)	
Neutropenia	30 (50)	8 (13)	7 (12)
Anemia	26 (43)	7 (12)	0
Thrombopenia	7 (12)	1 (2)	0
Toxicity-related data [*n* (%)]			
Blood transfusion	18 (30)		
EPO (secondary prophylaxis)	9 (15)		
G-CSF (primary prophylaxis)	4 (7)		
G-CSF required (secondary prophylaxis)	30 (50)		
Febrile neutropenia	6 (10)		
Hospitalisation due to infection	16 (27)		
Hospitalisation	26 (43)		
Deaths in association with AEs	4 (6,6)		

### PCC Efficacy

PCC achieved a 43.3% (i.e., 26 responses) ORR (95% CI: 30.8–55.8) with three complete responses and 23 partial responses. Thirteen patients experienced stable disease. DCR came out at 65% (i.e., 39 controlled patients) (95% CI: 52.9–77.1). Progression occurred in 16 patients (26.7%): seven patients experienced clinical progression but could not be radiologically evaluated, six patients experienced CT-scan-proved disease progression, and three patients showed dissociated responses with appearance of new metastases despite partial responses on target lesions. Five patients were not evaluable because of nonmeasurable lesions (**Table 5**). The ORR is similar in the 38 patients with a locoregional relapse in a previously irradiated area; in this population, we observe 14 responses, i.e., a response rate of 36.8%. Among the 14 patients for whom the cisplatin free interval was less than 6 months, we observed three partial responses.

**Table 5:** Efficacy after 8 weeks of treatment.

	*n* = 60
Overall response rate (95% CI)	43.3% (30.8–55.8)
Complete response	3 (5%)
Partial response	23 (38.3%)
Disease control rate (95% CI)	65% (52.9–77.1)
Stable disease	13 (21.7%)
Progressive disease	16 (26.7%)
Non evaluable	5 (8.3%)

**Figure 2:**
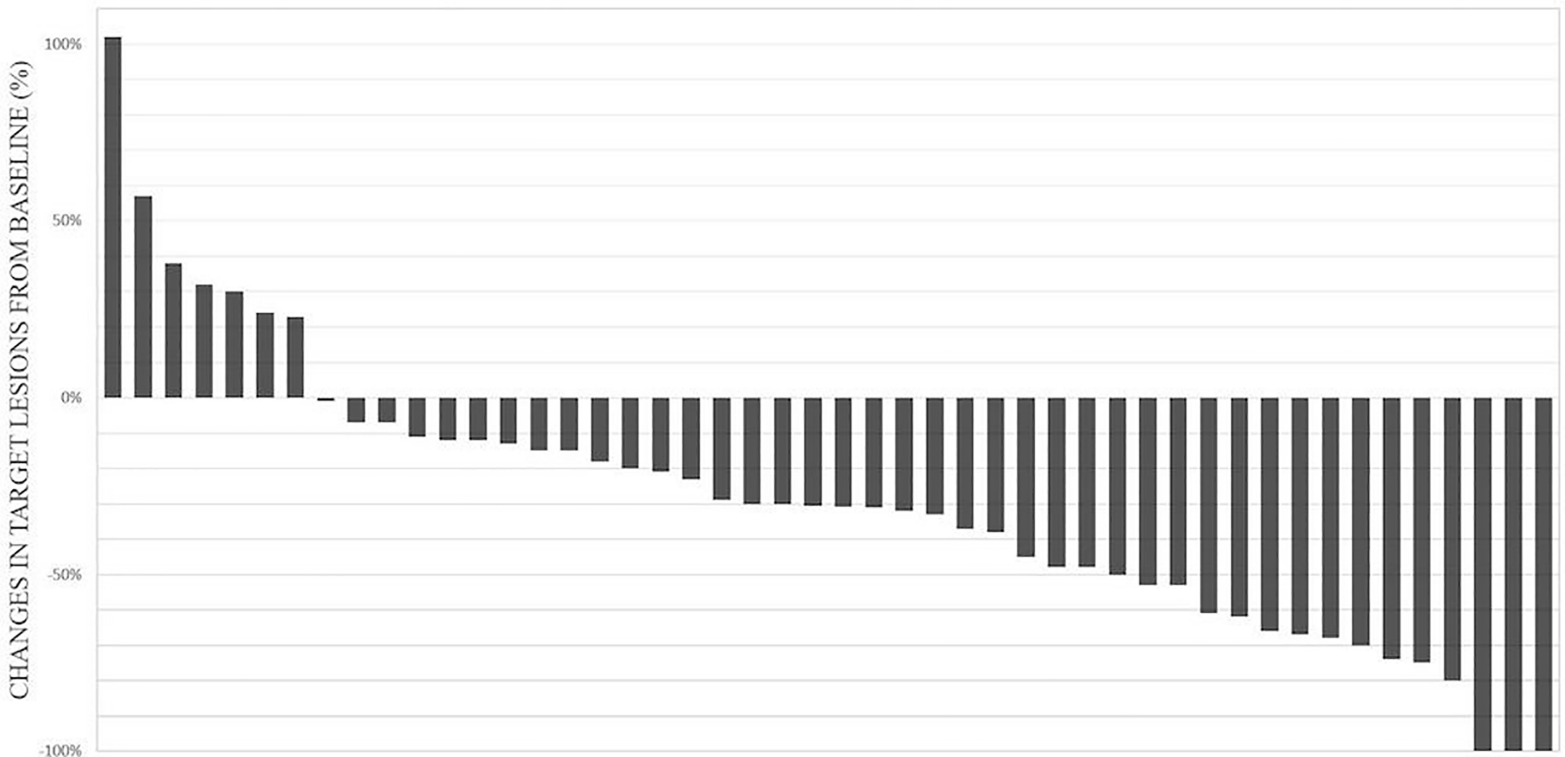
Waterfall plot of 48 assessable patients for change in target lesions.

Change in target lesions was not evaluable in 12 patients: five patients died before evaluation (four due to progressive disease, one due to infection); one patient was not compliant; one patient could not be re-evaluated due to clinical deterioration; and five patients did not have any measurable lesion according to RECIST criteria. Change in target lesions is shown in **Figure 2**.

**Figure 3:**
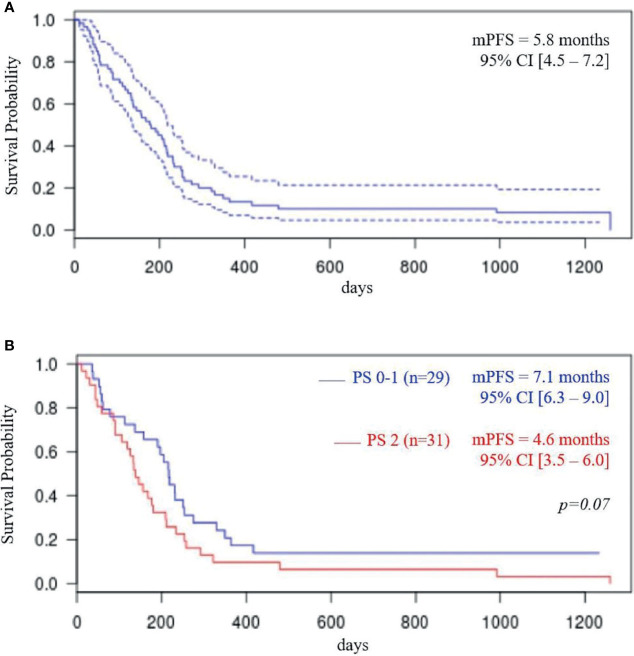
Progression-free survival (PFS) total population **(A)**; PFS according to performance status (PS) **(B)**.

**Figure 4:**
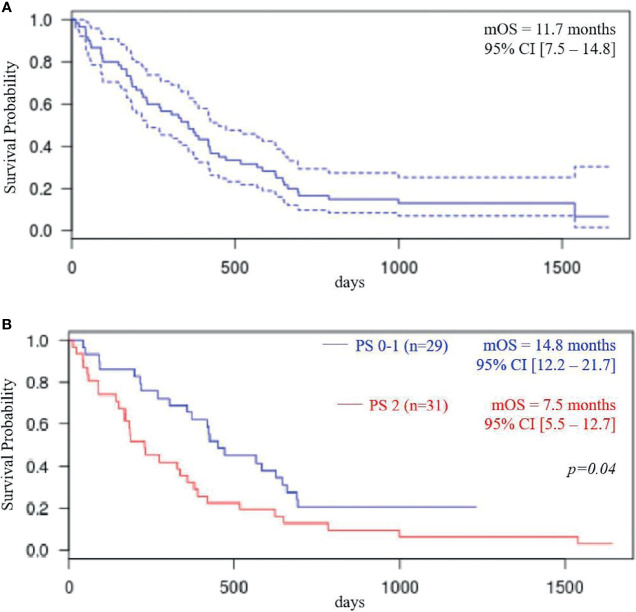
Overall survival (OS) total population **(A)**; OS according to performance status (PS) **(B)**.

With a median follow-up of 35.7 months (IQR 28.6–48.8), we observed a median OS of 11.7 months (95% CI: 7.5–14.8) and a median PFS of 5.8 months (95% CI: 4.5–7.2). Kaplan-Meier curve-line estimate of PFS and OS are shown in **Figures 3**, **4**.

In the ECOG-PS 0–1 population (i.e., 29 patients), median OS was 14.8 months (95% CI: 12.2–21.7) and median PFS was 7.1 months (95% CI: 6.3–9.0 months). In ECOG-PS 2 patients (i.e., 31 patients), median OS was 7.5 months (95% CI, 5.5–12.7; *p* < 0.04) and median PFS was 4.6 months (95% CI: 3.5–6.9; *p* = 0.07).

## Discussion

Our study shows that in first-line R/M HNSCC, a combination of paclitaxel, carboplatin, and cetuximab makes it possible to achieve results comparing favorably with what may be obtained through chemotherapies based on platinum-5-FU and cetuximab (3) or cisplatin-docetaxel and cetuximab (5, 6), and this, with particularly frail patients.

Indeed, in our study, 55 patients (92%) are cisplatin ineligible: ECOG-PS 2: 52%, platinum free interval <6 months: 23%, at least three frailty criteria: 62%, age ≥70: 17%; however, a 43.3% ORR, a 5.8-month median PFS, and a 11.7-month median OS are achieved.

Although a 11.7-month median OS compares favorably with the 10.1 months obtained through the EXTREME regimen (3), it seems however shorter than the 14.5 months observed with the TPEx (6) and the 14.7 months with the PCE regimens (9). It should be noted however that these two latter studies concern a more favorable population of patients ECOG-PS 0 or 1 and that patients who were enrolled into the TPExtreme study were cisplatin fit and under age 70. In our study, when we consider the ECOG-PS 0–1 patients, the 14.8 months median OS is very similar to that reported with the TPEx or PCE regimens.

Pêtre et al. reported on a weekly paclitaxel and carboplatin combination in a particularly frail and heavily pretreated population that produced a 40% ORR, a median PFS of 4.7 months, and a median OS of 9.1 months (11). Interestingly, this study confirms the major impact of cisplatin eligibility and ECOG-PS on survival outcomes: median OS is 13.7 months for cisplatin-eligible patients whereas it is only 8 months for cisplatin-ineligible patients. For cisplatin-ineligible patients, median overall survival decreases from 11.5 to 3.6 months in patients ECOG-PS 0–1 and ECOG-PS 2–3, respectively (11).

The number of administered weekly PCC cycles, cetuximab maintenance, and subsequent treatments seem to be important factors of survival. Indeed, in the retrospective study reported by Narveson et al, where treatment is limited to six weekly cycles of PCC, although a similar ORR of 37% is reported, median PFS is 4.6 months and median OS is only 5.25 months (12).

Weekly paclitaxel is a well-established regimen which allows high dose intensity with low hematologic toxicity (13). Likewise, fractionated administration of carboplatin allows also to decrease the hematologic toxicity and thus to maintain continuous weekly administration of chemotherapy with as few toxicity-related interruptions as possible (9, 11). Nevertheless, in our study, although toxicity is noteworthy, it is however mostly hematologic and may be managed. It is caused to a large extent by the frailty of the treated population. We observed 10% of febrile neutropenia, 50% of secondary prophylaxis using G-CSF, and 27% of hospital readmission for sepsis which resulted in four deaths (6.6%). It should be noted however that deaths in association with adverse events are only observed in ECOG-PS 2 patients. Likewise, in the TPExtreme study, a 7.7% rate of deaths in association with adverse events is reported in the EXTREME arm and 5.9% in the TPEx arm (6). We are now proposing G-CSF as a primary prophylaxis which significantly reduces infectious toxicity. Indeed, weekly administration of G-CSF is safe and effective as reported by Kies et al. in the first publication of the weekly PCC (10).

Results of our study like these observed with the TPEx and the PCE regimens as well as in the CETMET trial show that it is possible to replace advantageously 5-FU by a taxane. The CETMET trial is a randomized phase II study which shows that the replacement of 5-FU by paclitaxel allows to decrease toxicity: 60% of the grades III–V reported toxicities being in the EXTREME arm (*p* = 0.034). Moreover, authors observed an increasing trend in the median PFS from 4.37 months in the EXTREME arm to 6.5 months in the paclitaxel, carboplatin, and cetuximab arm (*p* = 0.064) (14). The randomized phase II study TPExtreme did not show however any survival advantage when compared with the EXTREME protocol but confirms a high median survival of 14.5 months and a favorable safety profile in the TPEx arm (6).

The EXTREME protocol has remained the standard of care for first-line R/M until 2019 when the KEYNOTE-048 study has demonstrated, as far as survival is concerned, the superiority of immunotherapy by the anti-PD-1 pembrolizumab for the CPS ≥1 population and of the combination of pembrolizumab, platinum, and 5-FU in all patients (15). Nevertheless, platinum and cetuximab associations may remain relevant as first- or second-line R/M treatments in situations such as described hereunder. As first-line treatment, the association of platinum and cetuximab is appropriate when using an anti-PD-1 which is not suitable because of insufficient efficacy whenever the combined positive score (CPS) is inferior to 1 (which in Europe precludes its use in association with chemotherapy as well) or in case the patient is considered ineligible for immunotherapy particularly with an active autoimmune disease treated by immunosuppressive agents. In first-line treatment, there remains a need to address the problem of fragile patient, particularly cisplatin unfit or ECOG-PS 2 patients for whom the toxicity of the pembrolizumab combined to platinum/5-FU is too severe to be considered (85% of grades III–IV). Pembrolizumab alone may be proposed (55% of grades III–IV toxicity, 17% related to treatment), but the ORR is only 19% for the CPS ≥1 population (15) which appears inappropriate for severely symptomatic patients (16), whereas the response rate provided by the weekly PCC regimen is 43%. Moreover, the efficacy of pembrolizumab for ECOG-PS 2 patients has not been formally studied. As a second-line R/M treatment, following the administration of pembrolizumab alone, platinum and cetuximab combinations remain perfectly relevant and this especially since increased efficacy of chemotherapy following the use of anti-PD-1 agents has been reported (17). The weekly PCC may be then an interesting option for cisplatin-unfit patient, even for those who are ECOG-PS 2.

Despite its two main advantages (response duration and low toxicity), immunotherapy by pembrolizumab alone benefits only a minority of patients, and the determination of PD-L1 by combined positive score (CPS) remains an imperfect predictive factor (23% of response for CPS >20). Moreover, progression rate at first evaluation is high (around 40%) which makes it risky to propose immunotherapy alone to severely symptomatic patients. Combining pembrolizumab to chemotherapy makes it possible to improve results (ORR 36%, median OS of 13 months). There remain however two drawbacks: high toxicity (85% grades III–IV) and when compared with EXTREME, a survival benefit which is not clearly demonstrated for all subgroups (CPS <20) (18).

Considering the above, it remains clearly necessary to improve the immunotherapy combination or the associated chemotherapy. The combination of weekly paclitaxel, carboplatin, and durvalumab which is intended specifically for frail patients in first-line R/M is presently being studied in the frail-immune trial (19).

Combining monalizumab with cetuximab in at least second-line R/M patients pretreated with platinum, 45% of whom had also received an antiPD-1, has shown promising results which still remain to be confirmed in a randomized phase II study (20). A probable synergy of an anti-PD-1 with cetuximab (21) would justify the next step of studying a combination of PCC with an anti-PD-1 or also PCC with monalizumab.

In addition, the PCC regimen showed promising results in the neoadjuvant setting with an ORR ranging from 70% to 97% (10, 22, 23). Haddad et al. showed in a phase II randomized study, in the neoadjuvant setting, that weekly PCC is as effective and less toxic than cetuximab–Taxotere/platin/5-FU (C-TPF) making weekly PCC an option of choice for TPF-unfit patients (24).

## Data Availability Statement

The raw data supporting the conclusions of this article will be made available by the authors, without undue reservation.

## Ethics Statement

Ethical review and approval was not required for the study on human participants in accordance with the local legislation and institutional requirements. Written informed consent for participation was not required for this study in accordance with the national legislation and the institutional requirements.

## Author Contributions

HC and CB conceptualized and drafted the study and the manuscript. MB and AJ critically reviewed the manuscript. HC, MB, RF, CF, and MK collected patients’ data. CB and PW reviewed all CT scans. AT realized statistical analysis. All authors contributed to the article and approved the submitted version.

## Conflict of Interest

CB is advisory board consultant for BMS and Astra Zeneca, and has received honoraria for consulting from Merck, BMS, Astra Zeneca and MSD. MB has received honoraria for consulting from BMS, Ipsen and MSD.

The remaining authors declare that the research was conducted in the absence of any commercial or financial relationships that could be construed as a potential conflict of interest.

## Publisher’s Note

All claims expressed in this article are solely those of the authors and do not necessarily represent those of their affiliated organizations, or those of the publisher, the editors and the reviewers. Any product that may be evaluated in this article, or claim that may be made by its manufacturer, is not guaranteed or endorsed by the publisher.
